# Editorial: The burden and impact of frailty in stroke

**DOI:** 10.3389/fneur.2026.2012685

**Published:** 2026-10-23

**Authors:** Piergiorgio Lochner, Simona Lattanzi, Andrea Morotti, Marialuisa Zedde

**Affiliations:** 1 Department of Neurology, University of the Saarland, Homburg, Saar, Germany; 2 Neurological Clinic, Department of Experimental and Clinical Medicine, Marche Polytechnic University, Ancona, Italy; 3 Department of Clinical and Experimental Sciences, University of Brescia, Brescia, Italy; 4 Neurology Unit, Stroke Unit, Azienda Unita Sanitaria Locale IRCCS di Reggio Emilia, Reggio Emilia, Italy; 5 Neurology and Stroke Unit, Ospedale del Mare, ASL Napoli 1 Centro, Naples, Italy

**Keywords:** Activities of daily living (ADL), C-reactive protein/albumin ratio (CAR), endovascular thrombectomy (EVT), Montreal cognitive assessment (MoCA) scores, re- existing cognitive impairment (PreCI), temporalis muscle thickness (TMT), intravenous thrombolysis (IVT)

Stroke is the second leading cause of death and the primary driver of adult disability worldwide. The global burden of cerebrovascular disease is undergoing a profound demographic shift, driven by an aging population (1, 2). Older age is a known risk factors for stroke, and both elderly and frailty are critical determinants of worse functional outcome, institutionalization, and mortality following an ischemic or haemorrhagic event (3).

As the rate of admissions for stroke in older people accelerates, defining the factors that govern response to treatment, particularly to acute reperfusion therapies like intravenous thrombolysis (IVT) and endovascular thrombectomy (EVT), becomes an urgent clinical need (4). Despite landmark advances in acute stroke therapy, current evidence-based stroke protocols remain inadequately validated in the oldest-old patients and in frail ones. In addition, age itself has an impact on frailty risk.

Unfortunately, chronological age alone offers poor resolution for clinical decision-making. Instead, the construct of frailty, a biological, neurocognitive, and social syndrome characterized by diminished physiological reserve, heightened vulnerability to stress, and multisystem dysregulation, has emerged as a key mediator of clinical recovery (5).

Frailty extends beyond chronological age, directly influencing post-stroke survival, functional recovery trajectory, and quality of life across the spectrum of cerebrovascular events. Yet, the interaction between premorbid multidimensional frailty, baseline blood-based markers, advanced radiomic profiles, and response to acute revascularisation strategies remains unexplored in everyday practice (6).

Frailty can be measured with different scales, each one with its own advantages and disadvantages depending on the cohort of application (4, 7).

Understanding the clinical impact of frailty is essential to optimize resource allocation, refine acute treatment algorithms, and build tailored post-stroke rehabilitation strategies.

This Research Topic brings together multi-center insights, novel biomarker discovery, machine learning models in radiomics, and clinical cohort data across pivotal studies to advance our understanding of prognostic modeling in elderly and frail stroke patients. It addresses pre-existing cognitive impairment (PreCI) and vulnerability in acute stroke care. PreCI frequently co-exists with physical frailty and vascular comorbidities, resulting in a complex clinical profile that might influence hyperacute decision-making and long-term recovery (Duan et al.).

Even though the age-standardized death rates and disability-adjusted life years rates declined over the 30 years, absolute stroke deaths and disability-adjusted life years continuously rose due to rapid population aging and metabolic disease expansion. A possible speculation is that PreCI correlates with greater baseline stroke severity, higher rates of acute complications, lower functional recovery, reduced survival, and a significantly higher rate of permanent institutionalization.

As part of the assessment of frail patients, it is important to evaluate certain biomarkers that can help determine their prognosis. One of these is sarcopenia. In this context, evaluating two retrospective cohorts (one with 152 ischemic stroke patients undergoing EVT and the other comprising 322 patients with spontaneous intracerebral hemorrhage) these two studies demonstrated a consistent pattern regarding temporalis muscle thickness (TMT). While TMT-assessed sarcopenia initially correlated with poorer functional outcomes (higher mRS scores) and higher mortality in unadjusted analyses across both groups, it lost statistical significance following multivariable adjustment. These congruent results could suggest that while cranial muscle thinning highlights a vulnerable patient phenotype, TMT-assessed sarcopenia functions primarily as a marker of advanced age and comorbidity burden rather than an independent driver of post-stroke disability (Bumann et al., Gubarevet et al.).

Baseline frailty, evaluated in one study through a 32-item Frailty Index), was not associated with 6-month cognitive recovery; instead, male sex, higher baseline Montreal Cognitive Assessment (MoCA) scores, and thalamic lesions were identified as independent predictors of favorable cognitive recovery (Duan et al.).

When looking for factors that can help to predict the prognosis of frail patients, one important issue is post-stroke dysphagia: it independently drives poor outcomes in the oldest-old, highlighting the critical value of frailty screening during hyperacute care planning (Sanner et al.).

Further interesting biomarkers are linked to the individual nutritional status and reserves, and the level of inflammation Of note, an elevated admission C-reactive protein/albumin ratio (CAR) independently predicted higher mortality in elderly patients with post-stroke dysphagia, though serum albumin alone offers comparable prognostic utility (Wang et al.).

Multivariable models can provide prognostic information in elderly stroke patients: in this regard, five independent risk factors (hemorrhagic stroke subtype, advanced age, hypoalbuminemia, elevated leukocyte count, and low activities of daily living (ADL) scores) were identified to predict pressure injuries during stroke recovery (Bi et al.).

Social frailty was significantly tied to ADL, depression, social support, language impairment, and stroke recurrence. To achieve an improvement in these patients, it seems necessary to highlight the need for targeted interventions focusing on depression management, ADL rehabilitation, and social support expansion.

In search of biomarkers that are correlated with prognosis, some authors have suggested that serum iron exhibits a threshold effect on post-stroke functional recovery, and that its correction may be critical for preserving independence in daily activities. Moreover, the hemoglobin-to-hematocrit ratio ratio exhibited a non-linear association with 3-month prognosis in acute ischemic stroke, highlighting the potential value of hemorheological balance monitoring for risk stratification (Bi et al.).

Finally, there are expectations for the application of artificial intelligence. For example, machine learning could be helpful to identify those patients who may not benefit from endovascular treatment (Hu et al.).

In conclusion, there are currently promising biomarkers and factors associated with the prognosis of frail stroke patients. To bridge remaining gaps in knowledge, future research must prioritize inclusive clinical trials, prospective multi-center registries, biomarker validation and advanced machine learning models evaluating acute effective revascularization response across stratified frailty, cognitive, and radiomic classes.

Translating these prognostic insights into dedicated clinical guidelines will ensure that therapeutic decisions in older stroke patients are evidence-based, equitable, and aligned with individual functional reserve.
